# Prophylactic titanium elastic nailing (TEN) following femoral lengthening (Lengthening then rodding) with one or two nails reduces the risk for secondary interventions after regenerate fractures: a cohort study in monolateral vs. bilateral lengthening procedures

**DOI:** 10.1186/1471-2474-14-302

**Published:** 2013-10-25

**Authors:** Frank Schiedel, Ulrich Elsner, Georg Gosheger, Björn Vogt, Robert Rödl

**Affiliations:** 1 Department of Children’s Orthopaedics, Deformity Correction and Foot Surgery, Münster University Hospital, Albert-Schweitzer-Campus 1, Münster, D-48149, Germany; 2 Department of General Orthopaedics and Tumour Orthopaedics, Münster University Hospital, Albert-Schweitzer-Campus 1, Münster, D-48149, Germany

**Keywords:** Limb lengthening, Regenerate fracture, Lengthening then rodding, TENs nailing after lengthening, Callus pattern

## Abstract

**Background:**

Femoral fracture rates of up to 30% have been reported following lengthening procedures using fixators. “Lengthening then rodding” uses one or two titanium elastic nails (TENs) for prophylactic intramedullary nailing to reduce this complication. The aim of the study was to decide if usage of only one TEN is safe or has it a higher risk of getting a fracture? And we asked if there is a difference between patients with monolateral or bilateral lengthening procedures according to their fracture rate?

**Methods:**

One or two TENs were implanted in two groups of patients (monolateral and bilateral) after femoral lengthening procedures. The regenerate quality was classified using the Li system and fractures were categorized using the Simpson and Kenwright classification. The follow-up period was at least 1 year after removal of the frame.

**Results:**

Sixty-seven patients with 101 femoral lengthening procedures were included in 2007–2011. Group A included 34 patients with bilateral lengthening due to congenital short stature. Group B consisted of 33 patients with congenital disorders with leg length discrepancies. Seven fractures in six patients were seen in group A and five fractures in group B. One patient had residual shortening of 1 cm, and 11 fractures healed without relevant deviation (< 5°) or shortening (< 5 mm). A soft-tissue infection in one patient led to early removal of one TEN.

**Conclusions:**

Fractures occurred in both groups of patients in total in 12 of the 101 cases (12%). The rate of secondary interventions was markedly reduced. Usage of one or two TENs did not influence the fracture rate.

## Background

Bone lengthening by callus distraction creates new bone over time. The procedure has been an established one ever since the importance of Ilizarov’s research was recognized. The wearing time of the fixator is the product of the length of the regenerate needed, the daily distraction rate, and the consolidation phase for the bone after distraction has been completed. On average, one month of fixator wearing time is estimated to be required for each centimeter of bone lengthening. Substantial fracture rates of up to 25% in the regenerate or the lengthened bone have been reported as a complication following the first few months after removal of the external fixators [1-4]. Fractures are seen particularly in cases of inappropriate accidents.

Systems for evaluating the quality of the callus have been developed, mainly using standard radiography, in order to calculate the appropriate time for removing the fixator [5-7]. Other methods using ultrasound, radiography, magnetic resonance imaging (MRI), computed tomography (CT), and dual-energy X-ray absorptiometry (DXA) have been described for assessing the density and stability of the regenerate [8-14]. There is as yet no gold standard for these approaches in everyday clinical practice.

Classification systems have been proposed for categorizing the complication of regenerate fractures [15,16]. The amounts of lengthening attempted are now more modest, and high-complication procedures involving more than 5–6 cm are nowadays generally avoided [17-20]. Combination procedures such as lengthening over a nail are more frequently used. Following epiphyseal closure, fully implantable lengthening nails can now be used. The method of lengthening over a rod has been described [2,21], and more recently lengthening and plating in the tibia as well [22]. When bone lengthening in the femur is necessary, callotasis using monolateral fixators still represents the gold standard for treatment in children [1,17].

Placement of a long leg–hip-spica-cast may provide protection against fractures in children during the first few weeks. Protecting the regenerate for a period of months using a cast is not realistic; fractures have also been reported when casts were in place [23,24].

Leaving the fixator in place for longer periods may weaken the regenerate if it is not dynamised axially in a timely fashion [1,13,17]. During the consolidation period in the cortical bone, the axial stress protection of the fixator can lead to conversion to a thin, hourglass-shaped regenerate in the femur [13,25].

The idea of prophylactic stabilization with use of TENs represents a new treatment approach that can be used for removal of the fixator at a defined time point within a standardized treatment course. The objectives of the present large prognostic study was to investigate the possible complications and the treatment results that are observed using this method. There are many questions beside the question how many fractures occur. Is there a difference between patients with monolateral or bilateral lengthening procedures, mainly when using only one TEN in many cases with too thin bones? Has Li’s classification a predictive value to determine patient’s risk of getting a fracture? Hypothesis was that fractures after inserting a TEN do not lead to relevant changes in the bone length or bone axis. Further it was to analyze if there are possible infectious problems caused by the one-stage exchange from an external to an internal procedure? [26,27].

## Methods

All patients with completed femoral lengthening procedures who were treated in our university pediatric orthopeadics department over a period of 36 months — from January 2008 to December 2010 — were included in a single-center cohort study to investigate the therapeutic outcome with this specific form of treatment in two groups of consecutive patients. Full prospective design with randomized group building or leaving one group as non treatment group was not possible at the time of starting the observation. Group A was the group of patients with bilateral lengthening procedures mainly in congenital short stature. Group B was the group of patients with monolateral lengthening procedures mainly in congenital disorders like CSF or hemimelias.

At the time of fixator removal, prophylactic elastic intramedullary nailing with one or two TENs was carried out as a one-stage procedure. In smaller bones this is an exception from the original thinking of 3 point support in nailing femoral fractures in children. The underlying principle — lengthening, then rodding — has been described previously elsewhere [2,21]. This fundamental change in the treatment regimen used after femoral lengthening was made in this institution starting on January 2008. It was in compliance with the Helsinki Declaration and part of therapeutical freedom. Approval of local ethical review board of the Medical Association of Westphalia-Lippe was granted for retrospective epidemiological analysis of this study cohort at 1^st^ September 2011.

**Figure 1:**
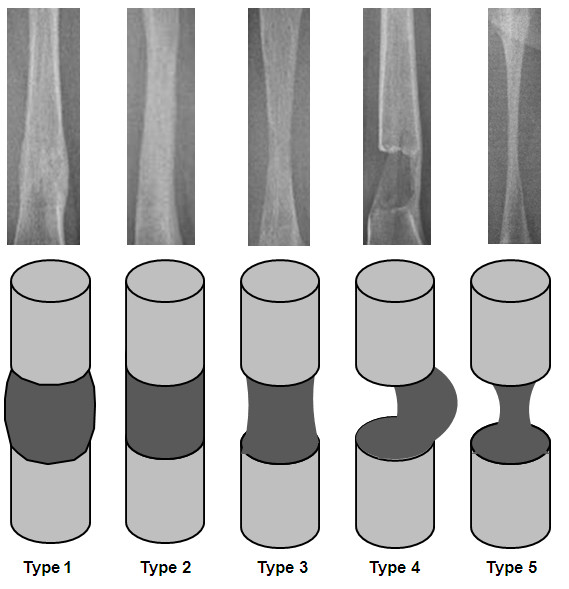
**Radiographic appearance of the five types of callus shape in the Li classification [**[12]**: 1, fusiform; 2, cylindrical; 3, concave hourglass shape; 4, incomplete, only lateral; and 5, only central, filiform.**

**Figure 2:**
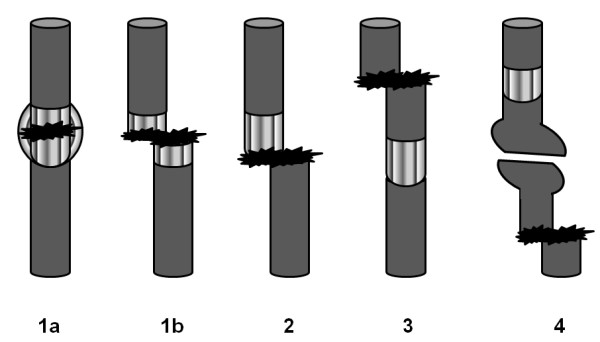
**The types of callus fracture or lengthening-associated fractures in the Simpson and Kenwright classification [**[15]**: Ia, bulging fracture in the regenerate; Ib, complete fracture in the regenerate; II, fracture at the bone–callus junction (host–regenerate junction); III, fracture in the lengthened leg outside the regenerate zone (pin site); IV, fracture in the lengthened bone outside the regenerate zone (not at a pin.**

Epidemiological data, complete medical file, and x-rays were available for the patients included. The minimum follow-up period was set as one year after removal of the TENs. The radiographic shape and density of the regenerate on the day before fixator removal were classified using the Li system (see also Figure 1 and Table 1) [12]. On the day of removal, the consolidated regenerate shape was again described in accordance with this scheme. For summary in Table 2 shape 1–3 were called stable and shape 4 and 5 unstable. Normal or intermediate density was called normal. All low (type 1,2,3 and 4) and intermediate sparse (type 5) and intermediate lucent (type 8) densities were called insufficient. The density types 6,7,9 and 10 were called sufficient. Any measurement was performed by four investigators (one resident and one senior consultant in radiology and one in pediatric orthopaedics) that were blind for the group building and the question of having a risk of fractures. Any complications of fixator treatment that had occurred in the meantime were classified in accordance with the Paley system as problems, obstacles, or complications [1]. Possible fractures in the prophylactic elastic nailed regenerates were described in accordance with the Simpson and Kenwright system (see Figure 2) [15].

**Table 1:** **The 10 types of regenerate quality observed on radiographic morphology, arranged by density based on the Li classification [**[12]**]**

	**Density**		
**Regenerate feature**	**Low**	**Intermediate**	**Normal**
Sparse	Type 1	Type 5	–
Homogeneous	Type 2	Type 6	Type 9
Heterogeneous	Type 3	Type 7	Type 10
Lucent	Type 4	Type 8	–

**Table 2:** Classification and important parameters for the clinical course in 12 of 101 lengthened femora – group A = seven cases in six bilateral lengthened patients, group B = five cases in monolateral lengthening procedures – in which fractures occurred after callotasis with TENs in place

**Group**	**Group A – bilateral**							**Group B - monolateral**				
**Case with fracture no.**	**1**	**2**	**3**	**4**	**5**	6*	7*	**8**	**9**	**10**	**11**	**12**
Amount of lengthening (mm)	58	60	59	61	67	60	60	43	25	48	10	30
Healing index (months/cm)	0.7	0.9	0.8	1.0	0.9	1.0	1.0	2.0	2.2	1.1	4.8	1.6
Li et al [12] types**												
removal of fixator	2–6	2–6	2–6	3–6	2–6	**5**–6	**5**-6	1–6	2–**2**	**4–2**	1–**2**	**4–2**
removal of TENs	1–6	1–9	2–9	2–9	2–9	1–9	1-9	2–9	2–9	4–9	1–10	2–10
At follow-up	2–9	2–9	2–9	2–9	2–9	2–9	2-9	2–9	2–9	2–9	1–10	2–10
Fracture type [15]	1b	1b	1b	1b	1b	1a	1a	1b	3	2	3	2
Fracture seen on day (after fixator removal)	0	0	41	41	50	22	22	28	0	46	0	0
Number of TENs used	2	2	1	1	1	1	1	2	2	2	2	2
TENs wearing time (days)	98	133	126	97	100	179	179	151	154	152	302	162

*Case 6 and 7 same patient, both sides fractured; ** read data a follows: regenerate feature: fusiform (type 1–5) – density: homogeneous intermediate (type 1–9); see also Table 1 and Figure 1.

### Surgical procedure

Following appropriate removal of the monolateral fixator and any superficial debridement of the pin tract required, TENs are inserted retrogradely in an aseptic procedure. Prophylactic TEN nailing represents a stable elastic osteosynthesis using metadiaphyseally positioned TENs for shaft fractures in patients in their growth years [28]. The common surgical procedure is implantation of two intramedullary opposing TENs with the same diameter. Only with this three-point support achieved for each implant, one provides an exercise-stable system that allows weight-bearing at an early stage — similar to the treatment provided for metaphyseal and diaphyseal fractures using this surgical procedure in patients in their growth years [23,24,28-30].

Implantation of only one TEN in smaller bones is a new concept. However in some cases of planned but not possible insertion of two TENs this surgical solution was used. Implantation is carried out via minimally invasive incisions outside of the pin tract, and the TENs are introduced just proximal to the distal femoral growth line, after opening of the medullary space with the awl under radiographic visualization. The tip of the TEN is slightly curved and beveled to allow better intramedullary navigation and to prevent the TEN from exiting the soft regenerate. Clamped into the Universal-T-Handpiece, the TEN is advanced — after gentle manual pre-bending, to allow better introduction — with delicate rotatory movements and carefully measured axial pressure. At the transition from intact corticalized bone to the regenerate zone (the host–regenerate junction), increased pressure is needed for advancement, and the curved tip of the TEN has to be pushed very precisely through the center of the regenerate. This has to be checked repeatedly with radiography at two levels.

The intention was to leave the rods three months in place at least to respect their further growing and the possible incorporation into the bone with secondary need of chiseling near to the physis. This was also choosen to address patients with pain and protrusion of the nail end into the moving soft tissues near to the knee joint. Nail end cups were not used. The TENs are removed with the patient under brief general anesthesia, often as an outpatient procedure. No additional external stabilization with a cast is required.

During this period, as well as in the fixator wearing period, full axial weight-bearing is permitted. In often very young patients who require leg lengthening, the early weight-bearing that is possible with this method, as well as the fact that there is no need for immobilization, are extremely important aspects of the treatment Even in cases with only one implantable TEN full weight bearing was allowed in contradiction to the idea of three point support only with two opposite TENs. TENs are available with a length of 450 mm and with the following diameters: 2.0, 2.5, 3.0, 3.5, and 4.0 mm.

For all cases in which implanting of TENs would not be surgically possible, the intention was to fall back on a hip-spica long–leg cast. In cases of occurrence of intraoperative fractures in the regenerate, treatment with TENs and removal of the fixator or leaving it in place, depending on the situation, was planned. Both scenarios were not seen in real.

Studies in trauma patients have shown that complications resulting from an increased risk of infection during a one-stage change from an external to an internal procedure are not to be expected with changes within the first 2 weeks [26,27]. Hardly any data are available for late changes of procedure after distraction of the callus in the fixator for a period of weeks or months. A similar method using static intramedullary nailing after lengthening, known as “lengthening then nailing” (LATN), is an effective procedure [21]. For lengthening procedures at the lower leg the concept of lengthening and plating (LAP) seems to be a new approach [22]. The results show that the fracture rate can be reduced, with a reduced fixator wearing time and faster bone healing. There is a risk of infection, but it is minimal, as there is no contact between the pin tract and the insertion sites for the nail.

### Statistics

Statistical analysis was carried out using Microsoft Excel. A statistician was entrusted with checking of the raw data material. Descriptive statistics stating percentage distributions were sufficient. The parameters age, fixator wearing time, healing index, usage of one or two TENs were in nonparametric Mann–Whitney-U test checked for their influence of getting a fracture.

## Results

67 consecutive patients (32 male, 35 female) underwent prophylactic surgical implantation of TENs at the time of fixator removal after bone lengthening in 101 femora. The underlying diagnoses are listed in Table 3. In the bilateral group (A) 34 patients and in the monolateral group (B) 33 patients were included.

**Table 3:** Diagnoses in 67 patients with femoral lengthening and prophylactic nailing on removal of the fixator

**Group**	**Diagnosis**	**Male**	**Female**	**Total**
		**(n)**	**(n)**	**(n)**
A	Achondroplasia	17	15	32
	Hypochondroplasia	0	2	2
B	Congenital disorders, hemimelia	11	9	20
	acquired LLD	1	6	7
	Other causes and idiopathic LLD	3	3	6
Sum		32	35	67

Group A includes 34 patients with 68 bilateral femoral lengthening procedures, group B includes 33 patients with unilateral lengthening procedures of the femur.

LLD, leg length discrepancies.

Twelve courses were associated with fractures as genuine complications. Seven of the fractures listed in Table 2 (cases no. 1–7) were observed in group A (bilateral). The remaining five fracture cases (Table 2, no. 8–12) were seen in the group B. One patient (fracture cases 6 and 7 in Table 2) had both-sided fractures. Table 3 Table 2.

In 47/68 cases in group A and two cases in group B only one TENs was used because of a too thin diameter of the bone. 6/7 cases with fractures in group A had one TENs, one patient with a 1b fracture had two 2.0 mm TENs. All five fracture cases in group B had two TENs with the usual three point fixation (the used diameters were 2.5 mm (1), 3.0 mm (2) and 3.5 mm (2)). Choosing of one or two TENs did not influence the occurrence of fractures, McNemar test in both groups was not significant.

There were other slight differences between the two groups.

The average age at surgery was 5.4 years (range 2.4–14.2 years) with a median of 4.7 years in the group of 34 patients with bilateral lengthening, compared with 8.8 years (range 3.6–16.6 years) with a median of 8.6 years, in the group of 33 patients with monolateral lengthening. Age did not influence the incidence of a fracture (p = 0.385 in group A, p = 0.190, CI level = 95).

The average lengthening in the bilateral group, at 60.4 mm (range 50–70 mm) with a median of 60 mm, was greater than that in the unilateral group, at 39.1 mm (range 10–60 mm) with a median of 40 mm.

The fixators had previously been worn for an average of 174 days (range 112–421 days, median 167 days) in group A and for an average of 154 days (range 70–283 days, median 152 days) in group B.

The healing index (time from osteotomy to removal of fixator in months per lengthened centimeter) in the bilateral group, at an average of 0.96 months/cm was faster than that in the unilateral group, averaging 1.53 months/cm. Fracture incidence was not influenced by that index in both groups (group A p-value = 0.855, group B p-value = 0.509).

The TENs remained in situ up to removal for an average of 115 days (range 15–302 days, median 106 days).

The final follow-up examination with radiography took place on average 11.4 months (range 6.1–32.4 months, median 9.2 months), after removal of the TENs and at least one year after removal of the fixator.

A total of 72 problem-free courses in accordance with the Paley criteria [1] were observed. Knee problems due to painful jutting of an TEN were observed in five patients. These were treated with analgetic administration and physiotherapy. The problems resolved completely after removal of the TENs. Obstacles observed included loosening with dislocation of a TEN in two patients, as an intervention requiring anesthesia was needed in order to reposition the rod using secondary tapping. In one case, a soft-tissue infection made it necessary to remove the medial TEN on the right, while the laterally introduced rod was able to remain in place.

Only two courses counted as a major complication in accordance with the Paley classification [1], One was with shortening of more than 1 cm occurring after fracture healing. Table 2 shows the data of the patients with fractures observed during the course.

Eight fractures represented type I fractures in the regenerate zone, while two fractures were in host-regenerate junction zone and two outside of the regenerate, representing type 2 or 3 fractures in the Simpson and Kenwright classification [15]. Type IV fractures of the tibia were not seen in this cohort.

With regard to the shape of the regenerate in accordance with the Li classification [12], the cylindrical shape 2 was observed at the time of fixator removal in group A in 51 cases, the fusiform shape 1 in 6 cases, and a different shape in 11 cases, in the total of 68 regenerates. The density and pattern at this time point were described as homogeneous intermediate type 6 in 49 of the 68 regenerates (72%). Other homogeneous regenerates were seen in 15 callus segments.

In group B the cylindrical shape 2 was observed at the time of fixator removal in 17/33 cases, the fusiform shape 1 in 12/33 cases, and a different shape in 4 cases. The density and pattern at this time point were described as homogeneous intermediate type 6 in 15 of the 33 regenerates (46%). Other homogeneous regenerates were seen in 12 callus segments.

## Discussion

Prophylactic intramedullary nailing with TENs at the time of fixator removal was carried out in a large cohort of 101 consecutive patients who underwent 68 bilateral and 33 monolateral femoral lengthening procedures at a university pediatric orthopedics department.

The study noted 12 fractures, corresponding to a fracture rate of 12% — low in comparison with other studies that did not use the procedure presented here [1-5,17].

Due to the heterogeneity of the diagnoses, randomization into an intervention group and a control group (leaving without TENs) was not possible, and this should be mentioned as a major limitation even in a prognostic study of this type.

In 10 of the 12 fractures, healing without axial deviation (< 5°) or relevant shortening (< 10 mm) was observed, no further treatment following the completion of regular treatment was necessary, and the planned treatment goal was reached without residue in accordance with the Paley criteria [1]. The fractures represented minor complications. Healing of a fracture with loss of correction is regarded as a major complication, with permanent impairment.

Usage of one or two TENs did not influence the incidence of a fracture. This was surprisingly because it is in contradiction to the idea of three point support that is only possible with two opposite TENs. With this results one has not to be afraid of a higher rate of fractures or of secondary loss of length or axial deviation.

Five of the 12 fractures (42%) occurred in connection with removal of the fixator or due to indirect or direct manipulation at the callus during implantation of the rod. Seven fractures were confirmed on radiography with the TENs in place, at an average of 36 days (range 22–50 days) after fixator removal. The fact that manipulation of the bone during removal of the fixator may lead to fractures — in the same way that external manipulation and manipulation during pushing forward of the TENs can also lead to rip of the callus regenerate internally, leading to fracture — remains debatable.

**Figure 3:**
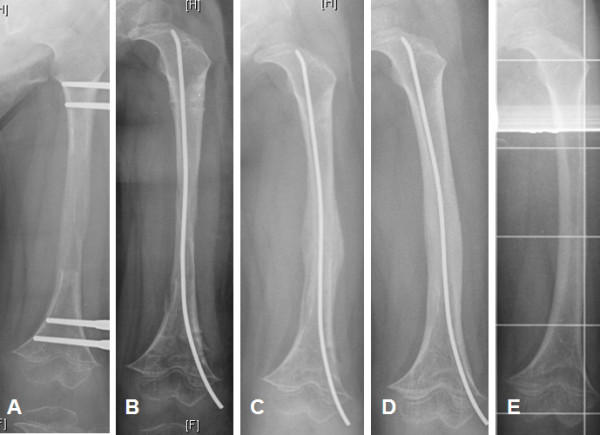
**A-D Radiographic examination and diagnosis of a fracture in the regenerate during treatment.** Boy (No 5 in Table 2) with femoral lengthening due to achondroplastic short stature, 5 years old: **A)** at the day of the removal of the fixator. **B)** day 0, after removal of the frame, one TEN was inserted due to prophylactic stabilization. **C)** x-ray at day 50 after removal shows a new callus formation within the regenerate, it must have been a fracture type 1b weeks ago, estimated caused by manipulation during insertion of the TEN, no other reason was applicable. **D)** 4 months (day 133) after removal of the fixator, before removal of the TEN with bony healing without loose of length or development of further malformation. **E)** late follow up, 1 year after removal of the TEN.

The radiographic findings of one those patients with a fracture Type 1b, seen at day 50 after removal of fixator are shown in Figures 3A - 3D (case no. 5 in Table 2). Tearing of the still-soft tube of callus due to inadvertent extrusion of the rod during implantation may be sufficient here to cause an excessive callus reaction, again with fusiform bone corresponding to initial bone healing, on later radiographs. To prevent the rod from exiting the regenerate, it is recommended that the hammer should not be used. Using only delicate, alternating slight quarter-rotations from the wrist, advancement of the rod can be achieved with the universal-T-handpiece when consistent pressure is applied, without the rod exiting from the soft regenerate Figure 3.

Usage of Li’s classification for description of the callus pattern seems not to be a reliable predictor of fractures. In group A all seven patients with fractures had sufficient density of the callus at the time of fixator removal. Only 2/7 fracture cases had a hourglass (unstable) shaped regenerate. In group B 3/5 fracture cases had a stable callus formation, but only one case had a sufficient density at the time of fixator removal.

A soft-tissue infection in one case occurred and made it necessary to remove the medial rod only 15 days after implantation, while the laterally introduced rod was able to remain in place. This case would represent an infection rate of lower than 0.7% of the TENs (1 of 153 implanted rods was to remove due to this reason) or 1% (1/101 bones) of the procedures or 1.5% of the patients (1/67 patients) in this study cohort. There is no general problem of infection due to the one-stage change from an external to an internal procedure, and infection need not be feared even after longer periods.

Lengthening then rodding is a new treatment protocol in femoral bone lengthening, which is capable of protecting the patient against fractures and secondary axial deviation for several months even after the fixator has been removed. There are no differences in patients with bilateral or monolateral procedures. Usage of one or two TENs does not influence the incidence of a fracture.

## Conclusion

Lengthening then rodding is a new treatment protocol in femoral bone lengthening, which is capable of protecting the patient against fractures and secondary axial deviation for several months even after the fixator has been removed. The appearance of the regenerate using the Li classification scheme is not a predictive value for the probability of a fracture after frame removal. All fractures healed with the previously implanted TEN; repeat surgery due to a fracture was not required. Healing with relevant loss of correction of > 1 cm only occurred in one case. In one case (1%), premature removal of a TEN only 15 days after implantation was necessary due to soft-tissue infection. There is no general problem of infection due to the one-stage change from an external to an internal procedure, and infection need not be feared. Only single shot antibiotics during surgery were administered routinely.

## Abbreviations

CSF: Congenital short femur; LAP: Lengthening and plating; LATN: Lengthening and then nailing; TEN: Titanium elastic nail.

## Competing interests

The authors declare that they have no competing interests.

## Authors’ contributions

FS conceived of the study, FS and GG did the conception and design of the study. FS and RR performed most of the surgeries. UE and BV had acquired the data and participated in most of the surgeries, UE, BV and RR did the analysis of the x-rays. FS, BV performed the analysis and interpretation of data. FS performed the statistical analysis, UE, GG and RR helped to draft the manuscript. GG supported the revision of the manuscript and all authors read and approved the final manuscript.

## Pre-publication history

The pre-publication history for this paper can be accessed here:

http://www.biomedcentral.com/1471-2474/14/302/prepub
